# Four-Quadrant Facial Function in Dysphagic Patients after Stroke and in Healthy Controls

**DOI:** 10.1155/2014/672685

**Published:** 2014-03-04

**Authors:** Mary Hägg, Lita Tibbling

**Affiliations:** ^1^Department of Otorhinolaryngology, Speech & Swallowing Centre, Hudiksvall Hospital, 824 81 Hudiksvall, Sweden; ^2^Research & Development Centre, Uppsala University/Gävleborg, 751 85 Uppsala, Sweden; ^3^Department of Otorhinolaryngology, Linköping University, 581 85 Linköping, Sweden

## Abstract

This study aims to examine any motility disturbance in any quadrant of the face other than the quadrant innervated by the lower facial nerve contralateral to the cortical lesion after stroke. Thirty-one stroke-afflicted patients with subjective dysphagia, consecutively referred to a swallowing centre, were investigated with a facial activity test (FAT) in all four facial quadrants and with a swallowing capacity test (SCT). Fifteen healthy adult participants served as FAT controls. Sixteen patients were judged to have a central facial palsy (FP) according to the referring physician, but all 31 patients had a pathological FAT in the lower quadrant contralateral to the cortical lesion. Simultaneous pathology in all four quadrants was observed in 52% of stroke-afflicted patients with dysphagia; some pathology in the left or right upper quadrant was observed in 74%. Dysfunction in multiple facial quadrants was independent of the time interval between stroke and study inclusion. All patients except two had a pathological SCT. All the controls had normal activity in all facial quadrants. In summary the majority of poststroke patients with dysphagia have subclinical orofacial motor dysfunction in three or four facial quadrants as assessed with a FAT. However, whether subclinical orofacial motor dysfunction can be present in stroke-afflicted patients without dysphagia is unknown.

## 1. Introduction

In the acute period after a stroke attack 35–75% of the patients suffer from oropharyngeal dysphagia [1] with misdirected swallowing and aspiration. The frequency of dysphagia in patients surviving the first few weeks after a stroke is about 10% [2]. In clinical practice, we have noticed that motor activity supplied by the upper branches of the facial nerve can be somewhat affected in stroke-afflicted patients with dysphagia which conflicts with common clinical practice, that central facial nerve palsy or lower motor neuron injury in stroke-afflicted patients is recognized as a contralateral palsy of the lower orofacial muscles [3, 4]. However, in an electromyography study, Yildiz et al. [3] have shown that the “healthy” side of the lower facial branch can also be subclinically affected. Since the facial nerve is part of the swallowing mechanism [5–7], our aim was to study whether motility in any of the four facial quadrants other than the quadrant contralateral to the cortical lesion is affected in patients with stroke and dysphagia. To this end, patients with stroke and subjective dysphagia were investigated with both a facial activity test (FAT) [8] and a swallowing capacity test (SCT) [9, 10].

## 2. Methods

### 2.1. Study Design

This is a prospective clinical study of the activity of the upper and lower facial quadrants in healthy control participants and in patients who have experienced a single stroke attack and subjective oropharyngeal dysphagia. The stroke afflicted patients were separated into three groups according to the median time interval between the stroke incident and the start of the study.

### 2.2. Study Population

The initial patient cohort comprised 38 patients with a history of a one-time stroke attack, subjective oropharyngeal dysphagia as assessed by physicians in a stroke ward and who were consecutively referred to a swallowing centre. The included patients did not have any other neurological disease than stroke. Seven of these 38 patients were excluded, 6 got a new stroke, and 1 was unable to cooperate. The final study included 31 patients (11 women and 20 men; median age 67 years; range 46 to 82 years) during the years 2006 to 2012. The patients were investigated a median of 6 months (range 2 days to 9 years) after a stroke attack, with a right-sided cortical lesion in 11 patients, a left-sided lesion in 18 patients, and bilateral lesions in 2 patients, as assessed with computerized tomography. Facial palsy at rest (FP), as assessed by the referred physicians, was observed in 8 patients on the left side, in 7 patients on the right side, and in 1 of the 2 patients with bilateral lesions. FP at rest was not present in 15 patients. The patients were separated into three groups according to the median time interval between the stroke incident and the start of the study (Tables 1, 2, and 3): Group A, 9 days (*n* = 11); Group B, 6 months (*n* = 7); Group C, 2 years (*n* = 13). The control group comprised 15 healthy participants (6 women and 9 men; median age 66 years; range 52 to 77 years).

### 2.3. Facial Activity Test

A FAT [8] including seven different exercises, each scored on a five-point scale, was used to measure the motility function of all four facial quadrants. The patients and controls were seated in an upright and slightly forward position and instructed to perform the following movements: upper facial branch: (1) close the eyes, (2) raise the eyebrows, and (3) wrinkle the eyebrows; lower facial branch: (4) wrinkle the nose, (5) pout the lips, (6) smile, and (7) repeat “oh-eeh” three times as quickly and rhythmically as possible. The score sum of each facial branch and each side was divided by the number of items. A score sum of zero = normal, 1 = mild dysfunction, 2 = moderate dysfunction, 3 = severe dysfunction, and 4 = total inability. The intra-/interrater reliability of scoring orofacial muscle function has been shown to have a high kappa coefficient of 0.90 [8].

### 2.4. Swallowing Capacity Test

For the SCT, each patient was instructed to sit upright and swallow 150 mL of water as quickly as possible without pausing. The time was recorded from onset of drinking until the final swallow was completed. The remaining water in the glass was measured. Swallowing capacity (SC) was defined as the amount of water swallowed divided by time and is expressed as milliliters per second (mL/s). When the patient was unable to swallow anything at all or began with an initial wrong-way swallowing when trying with a teaspoon of water one to three times, the SC was graded as zero. A SC of 10 mL/s is regarded as the lower limit of normal [9, 10]. The SCT has been demonstrated to have high intra-/interrater and test-retest reliability. It has been claimed to give reliable and valid index for assessing SC, disordered swallowing in neurological patients, and to be of value in monitoring therapeutic response [9, 10].

### 2.5. Data Collection

This study was designed according to good clinical practice (GCP). All data were collected according to an initial protocol.

### 2.6. Definitions

Facial palsy (FP) is defined as a visible dysfunction at rest in the lower facial branch contralateral to the cortical lesion. A subclinical facial dysfunction is defined as the inability to properly perform facial and periorbital muscle activities on demand in any facial quadrant.

### 2.7. Ethical Considerations

This study was approved by the local Ethics Committee for Human Research at the Uppsala Medical Faculty, Sweden (Dnr 2004: M-435). Written or verbal consent to take part in the study was obtained from all participants.

## 3. Results

Facial dysfunction in more quadrants than the quadrant expected to be affected (i.e., other than the quadrant innervated by the lower branch contralateral to the lesion) was observed in 100% of patients with a right-sided cortical lesion (*n* = 11), 83% of patients with left-sided cortical lesion (*n* = 18), and all patients with bilateral cortical lesions (*n* = 2; Tables 1–3). Some degree of simultaneous dysfunction in all four quadrants was observed in 52% of all patients (55% group A, 57% group B, and 46% group C; Tables 1–3). Some pathology in either the right or the left upper facial branch was observed in 74% of all patients. Patients with a right-sided cortical lesion (*n* = 11) had a mean FAT score of 1.5 (8 patients with FP), patients with a left-sided cortical lesion (*n* = 18) had a mean FAT score of 2.1 (7 patients with FP), and patients with bilateral lesions (*n* = 2) had a mean FAT score of 0.96 (one patient with FP). The lower facial quadrant contralateral to the one-sided cortical lesion showed FA dysfunction in all 29 patients. Patients with FP (*n* = 16) had a mean FAT score of 1.9. Patients without FP (*n* = 15) had a mean FAT score of 1.5. In all 29 patients with a unilateral cortical lesion the mean FAT score in the contralateral lower quadrant was 2.2 (range 0.3–4), contralateral upper quadrant 1.6 (range 0–4.0), ipsilateral lower quadrant 1.5 (range 0–4), and the ipsilateral upper quadrant 1.1 (range 0–3.7) (Tables 1–3). Mean SCT was 1.3 mL/s (range 0–7.1) in Group A, 3.2 mL/s (range 0−14.4) in Group B, and 3.3 mL (range 0–10.2) in Group C (Tables 1–3). There was no correlation between FAT score and the degree of dysphagia as assessed with the SCT. All the 15 controls exhibited normal findings (mean FAT score = 0) in all four quadrants and had a normal SCT.

## 4. Discussion

In more than one-half of patients with stroke and dysphagia, dysfunction of facial motility was present in all four quadrants. All patients had FA dysfunction in the lower facial branch contralateral to the cortical lesion, but a clinical diagnosis of FP was only given in half of the patients. For a long time, the classical (and prevailing) view has been that the neural links between cortex and brainstem give the upper motor neuron of the facial nerve a double cortical representation, while providing the lower motor neuron only contralateral representation. This view is not consistent with our findings. Bilateral motility dysfunction in the lower facial branch agrees with results shown in 2005 by Yildiz et al. [3], that corticobulbar innervations of the lower facial muscles are bilateral. Mahadevappa et al. [4] also questioned the classical conception that the lower facial muscles receive only unilateral innervation from the contralateral cerebral cortex. They even demonstrated that both the upper facial nucleus and the lower facial nucleus receive bilateral cortical projections in monkeys. Morecraft et al. [11] pointed to a historic trail of clinical observations that indicates a higher order regulation of facial expression, both voluntary and emotional, that does not occupy a specific site in the brain or manifest through a single neural projection system. This higher order regulation may indicate that activity on demand is dependent on correct information from both cortical sides and may explain our findings of bilateral dysfunction in the lower as well as in the upper facial quadrants. Dysfunction in all facial quadrants will likely also have emotional implications owing to the face's inability to express feelings [12].

Scoring or grading of facial function in other studies has either focused on the lower branch of the facial nerve in connection with central brain lesions [13] or on peripheral facial palsy with one-sided lesions, as assessed with electromyography [14]. The FAT [8] scoring (on which control participants scored normally in the present study) was constructed to cover all motor end points of the facial nerve in cerebral lesions. The advantages of this test in clinical settings are that it has been shown to have a high kappa coefficient regarding intra-/interrater reliability [8] and is easily accessible, inexpensive, and independent of technical equipment. It is not possible to evaluate whether dysfunction of the facial nerve is involved in impaired swallowing capacity in this study since subjective dysphagia was one of the inclusion criteria for the study. It must be emphasized that nothing can be said about facial dysfunction in patients without oropharyngeal dysphagia, because the facial nerve is involved in both swallowing function and facial activity.

## 5. Conclusion

It may be expected that the majority of post-stroke patients with dysphagia will have a subclinical motor dysfunction in three or four facial quadrants, even without the presence of a classical central one-sided palsy. It may also be expected that dysfunction can persist in several facial quadrants for years after stroke. However, it remains unknown whether motor dysfunction in several facial quadrants can be present in stroke-afflicted patients without dysphagia.

## Figures and Tables

**Table 1:** Four-quadrant (upper, lower, right, and left) facial function in Group A (status days after stroke). Individual data are given for each patient with stroke and dysphagia (*n* = 11).

Patient ID *n* = 11	Interv (days)	Brain lesion type R	FP L	Brain lesion type L	FP R	Brain lesion type Bilateral	FAT 4 Quadr R/L	FAT 3 Quadr U + Low R/L	FAT 2 Quadr U + Low R/L	FAT 2 Quadr Low Bilateral	SCT mL/s
36	2	I	**X**				1.7 2.7 2.0 **3.0**				0

19	6			I			1.0 1.0 1.5 1.3				0

28	6	I	**X**						0 0.7 0 **1.5**		3.4

20	7		**X**			I	1.3 1.3 1.3 **1.8**				0

22	7	I	**X**							0 0 1.5 **2.8**	0

21	10	I	**X**						0 1.2 0 **1.3**		0

34	10			H	**X**		4.0 3.0 **3.5** 3.0				0

17	20	I	**X**						0 1.0 0 **1.5**		0

26	28	I						0 0.3 0.8 1.0			7.1

30	28			I			3.0 3.0 3.0 2.8				1.8

38	28			I	**X**		3.7 3.7 **4.0** 4.0				2.2

ID: identification number; Interv: time interval between stroke event and start of study; FP: facial palsy; FAT: score of facial activity test; R: right side; L: left side; Low: lower facial branch; U: upper facial branch; I: infarction; X and figures in bold: FP as assessed by a referring physician; H: hemorrhage; SCT: swallowing capacity test; Quadr: quadrants.

**Table 2:** Four-quadrant (upper, lower, right, and left) facial function in Group B (status months after stroke). Individual data are given for each patients with stroke and dysphagia (*n* = 7).

Patient ID *n* = 7	Interv (months)	Brain lesion type R	FP L	Brain lesion type L	FP R	Brain lesion type Bilateral	FAT 4 Quadr R/L	U + Low R/L	FAT 2 Quadr U + Low R/L	FAT 2 Quadr Low Bilateral	SCT mL/s
18	1.5			I	X		2.8 2.7 **4.0** 4.0				0

8	3	I	**X**				1.3 2.7 1.3 **3.3**				0

2	6	I	X					0 0.7 1.0 **1.0**			0

13	6			I			3.3 3.3 3.0 2.5				0

10	6			I						0 0 3.0 3.0	14.4

12	7			H			3.3 1.0 3.5 1.8				0

1	9	I	**X**					0.3 0.3 0.0 **2.0**			7.9

ID: identification number; Interv: time interval between stroke event and start of study; FP: facial palsy; FAT: score of facial activity test; R: right side; L: left side; Low: lower facial branch; U: upper facial branch; I: infarction; X and figures in bold: FP as assessed by a referring physician; H: hemorrhage; SCT: swallowing capacity test; Quadr: quadrants.

**Table 3:** Four-quadrant (upper, lower, right, and left) facial function in Group C (status years after stroke). Individual data are given for each patient with stroke and dysphagia (*n* = 13).

Patient ID *n* = 13	Interv (years)	Brain lesion type R	FP L	Brain lesion type L	FP R	Brain lesion type Bilat.	FAT 4 Quadr R/L	FAT 3 Quadr U + Low R/L	FAT 2 Quadr U + Low R/L	FAT 2 Quadr Low Bilat.	FAT 1 Quadr R/L	SCT mL/s
3	1	H					3.3 3.3 3.0 3.3					0

27	1			I							0 0 0.8 0	10.2

33	1			I			3.3 2.3 3.0 1.5					7.2

7	1.2			I			1.7 1.7 2.5 2.5					2.8

32	1.5			I	**X**						0 0 **0.3** 0	1.6

5	2			I			2.0 1.3 1.8 1.5					4.8

35	2.2			I			1.3 1.3 2.5 0.8					0.6

9	3.5			H						0 0 1.8 1.3		4.0

37	5			I	**X**					0 0 **1.3** 0.3		2.2

29	5			I	**X**		3.7 2.3 **3.8** 3.5					0.5

6	5.3	H								0 0 0.5 0.5		0

11	5.5					I		0 0.7 0.3 0.5				0

15	9			I	**X**						0 0 **0.3** 0	9.1

ID: identification number; Interv: time interval between stroke event and start of study; FP: facial palsy; FAT: score of facial activity test; R: right side; L: left side; Low: lower facial branch; U: upper facial branch; I: infarction; X and figures in bold: FP as assessed by a referring physician; H: hemorrhage; SCT: swallowing capacity test; Quadr: quadrants.

## References

[1] Sorensen RT, Rasmussen RS, Overgaard K, Lerche A, Johansen AM, Lindhardt T (2013). Dysphagia screening and intensified oral hygiene reduce pneumonia after stroke. *Journal of Neuroscience Nursing*.

[2] Broadley S, Cheek A, Salonikis S (2005). Predicting prolonged dysphagia in acute stroke: the Royal Adelaide Prognostic Index for Dysphagic Stroke (RAPIDS). *Dysphagia*.

[3] Yildiz N, Ertekin C, Ozdemirkiran T (2005). Corticonuclear innervation to facial muscles in normal controls and in patients with central facial paresis. *Journal of Neurology*.

[4] Mahadevappa K, Vora A, Graham A, Nesathurai S (2010). Facial paralysis: a critical review of accepted explanation. *Medical Hypotheses*.

[5] Hägg M, Anniko M (2010). Influence of lip force on swallowing capacity in stroke patients and in healthy subjects. *Acta Oto-Laryngologica*.

[6] Hägg M, Olgarsson M, Anniko M (2008). Reliable lip force measurement in healthy controls and in patients with stroke: a methodologic study. *Dysphagia*.

[7] Hägg M, Anniko M (2008). Lip muscle training in stroke patients with dysphagia. *Acta Oto-Laryngologica*.

[8] Hägg M, Larsson B (2004). Effects of motor and sensory stimulation in stroke patients with long-lasting dysphagia. *Dysphagia*.

[9] Nathadwarawala KM, Nicklin J, Wiles CM (1992). A timed test of swallowing capacity for neurological patients. *Journal of Neurology Neurosurgery and Psychiatry*.

[10] Kalyanee M, Nathadwarawala KM, McGroary A, Wiles CM (1994). Swallowing in neurological outpatients: use of a timed test. *Dysphagia*.

[11] Morecraft RJ, Stilwell-Morecraft KS, Rossing WR (2004). The motor cortex and facial expression: new insights from neuroscience. *Neurologist*.

[12] Konecny P, Elfmark M, Urbanek K (2011). Facial paresis after stroke and its impact on patients’ facial movement and mental status. *Journal of Rehabilitation Medicine*.

[13] Kimura J (2006). Electrodiagnosis of the cranial nerves. *Acta Neurologica Taiwanica*.

[14] Yildiz S, Bademkiran F, Yildiz N, Aydogdu I, Uludag B, Ertekin C (2007). Facial motor cortex plasticity in patients with unilateral peripheral facial paralysis. *NeuroRehabilitation*.

